# The association between neuroendocrine/glucose metabolism and clinical outcomes and disease course in different clinical states of bipolar disorders

**DOI:** 10.3389/fpsyt.2024.1275177

**Published:** 2024-01-24

**Authors:** Xu Zhang, Yaling Zhou, Yuexin Chen, Shengnan Zhao, Bo Zhou, Xueli Sun

**Affiliations:** ^1^Sichuan Provincial Center for Mental Health, Sichuan Provincial People’s Hospital, School of Medicine, University of Electronic Science and Technology of China, Chengdu, China; ^2^Key Laboratory of Psychosomatic Medicine, Chinese Academy of Medical Sciences, Chengdu, China; ^3^The Fourth People’s Hospital of Chengdu, Chengdu, China; ^4^Mental Health Center, West China Hospital of Sichuan University, Chengdu, China

**Keywords:** bipolar disorder, HPA axis, HPT axis, glucose metabolism, insulin resistance, bipolar depression, bipolar manic, treatment outcomes

## Abstract

**Objective:**

The treatment of bipolar disorder (BD) remains challenging. The study evaluated the impact of the hypothalamic–pituitary–adrenal (HPA) axis/hypothalamic–pituitary-thyroid (HPT) axis and glucose metabolism on the clinical outcomes in patients with bipolar depression (BD-D) and manic bipolar (BD-M) disorders.

**Methods:**

The research design involved a longitudinal prospective study. A total of 500 BD patients aged between 18 and 65 years treated in 15 hospitals located in Western China were enrolled in the study. The Young Mania Rating Scale (YMRS) and Montgomery and Asberg Depression Rating Scale (MADRS) were used to assess the BD symptoms. An effective treatment response was defined as a reduction in the symptom score of more than 25% after 12 weeks of treatment. The score of symptoms was correlated with the homeostatic model assessment of insulin resistance (HOMA-IR) index, the HPA axis hormone levels (adrenocorticotropic hormone (ACTH) and cortisol), and the HPT axis hormone levels (thyroid stimulating hormone (TSH), triiodothyronine (T3), thyroxine (T4), free triiodothyronine (fT3), and free thyroxine (fT4)).

**Results:**

In the BD-M group, the YMRS was positively correlated with baseline T4 (*r* = 0.349, *p* = 0.010) and fT4 (*r* = 0.335, *p* = 0.013) and negatively correlated with fasting insulin (*r* = −0.289, *p* = 0.013). The pre-treatment HOMA-IR was significantly correlated with adverse course (*p* = 0.045, OR = 0.728). In the BD-D group, the baseline MADRS was significantly positively correlated with baseline fT3 (*r* = 0.223, *p* = 0.032) and fT4 (*r* = 0.315, *p* = 0.002), while baseline T3 (*p* = 0.032, OR = 5.071) was significantly positively related to treatment response.

**Conclusion:**

The HPT axis and glucose metabolism were closely associated with clinical outcomes at 12 weeks in both BD-D and BD-M groups. If confirmed in further longitudinal studies, monitoring T3 in BD-D patients and HOMA-IR for BD-M could be used as potential treatment response biomarkers.

## 1 Introduction

Bipolar disorder (BD) is a severe chronic mental disorder with a lifetime prevalence ranging between 0.6% and 2.5% (1, 2). The disease is characterized by two different clinical states: depressive lows (BD-D) and manic or hypomanic highs (BD-M). BD is a debilitating condition that negatively impacts the quality of life of patients, and many BD patients have suicidal tendencies. The treatment of BD generally involves a combination of medication, psychotherapy, and lifestyle adjustments. However, the treatment of BD remains challenging and patients will require long-term treatment to prevent relapses of depression or mania. The selection of the optimal pharmacological treatment has important implications on clinical outcomes, mood stabilization, and restoration of normal metabolic and nervous system functions (3). However, these medications may also have side effects. Therefore, there is a need to identify potential biomarkers to monitor treatment response and, hence, select the optimal therapy for the patient.

Previous studies have found an association between neuroendocrine abnormalities and the development of affective disorders. Particularly, dysfunctions of the hypothalamic–pituitary–adrenal (HPA) axis and the hypothalamic–pituitary-thyroid (HPT) axis have been linked with the development of BD (4–6)_._ The HPA axis regulates the body’s response to stress by releasing cortisol (5–7). On the other hand, the HPT axis controls the production of thyroid hormones crucial for metabolism and overall health. Diseases that cause HPA axis dysfunction can dysregulate the release of corticotropin-releasing hormone (CRH) and alter the HPT axis regulation (8, 9). Studies have shown that patients with rapid cycling BD are more likely to have thyroid abnormalities, such as hypothyroidism, than those with unipolar depression (4, 10–14).

In addition to thyroid dysfunction, BD patients often present with insulin resistance and altered glucose metabolism. Studies have found common genome-wide and locus-specific genetic overlap patterns between BD patients and those suffering from type 2 diabetes mellitus (T2DM) (15). T2DM is mainly characterized by the development of tissue resistance to insulin. Studies have shown that these two disorders may share the same etiopathogenetic mechanisms that cause alterations in insulin signaling and glucose metabolism (15, 16). Meta-analyses and cohort studies also found an association between mood disorders and T2DM (17). However, although no longitudinal studies investigated the relationship between T2DM and long-term treatment outcomes of patients with BD, a Danish register-based cohort study found temporally ordered associations between BD and T2DM (18). Insulin signaling may play an important role, because of the significant genetic covariance found between BD and T2DM/metabolic syndrome within gene sets related to insulin signaling when using the largest available genome-wide association studies data and exploring comorbidity at the gene set/pathway level (19). Moreover, certain neurochemical and neuroanatomical changes commonly found in BD patients, such as reduced n-acetyl aspartate (NAA) levels in the prefrontal lobe and reduced hippocampal volume, have also been associated with impaired glucose metabolism (20, 21).

Studies have shown that even mild thyroid dysfunction might play an important role in the treatment response (22), risk of readmission (14), and clinical outcomes in BD patients (4). Altered glucose metabolism has also been reported in BD patients (23). The severity of the metabolic dysfunction in BD patients was linked with a worse prognosis and higher incidence of adverse events, such as cardiovascular events (24). However, the dysfunctions within the hormones regulated by the HPA–HPT axis and glucose metabolism (especially insulin resistance) on disease progression, and treatment response in patients with BD remain unclear. Therefore, a longitudinal study is necessary to assess whether changes in the HPA-HPT axis and glucose metabolism could be used as biomarkers for treatment response.

This study aimed to investigate the impact of changes in the HPA and HPT functional axis and glucose metabolism in BD patients in relation to healthy individuals. In addition, we also evaluated the association between the HPA, HPT, and glucose metabolism dysfunction indices on treatment outcomes in patients with BD-M and BD-D.

## 2 Materials and methods

### 2.1 Research design

The study used a prospective, multicenter, longitudinal research design and was conducted at 15 clinical sites located in Western China from December 31, 2018 to December 31, 2019 (Appendix 1).

### 2.2 Data collection

Individuals aged between 18 and 65 years who received inpatient treatment at one of the 15 centers for depressive or manic episodes in BD as defined by ICD-10 (25) were eligible for the study. All patients were diagnosed independently by two trained senior psychiatrists. Patients who had cyclical BD episodes, were in remission for BD, had a history of alcohol or substance dependence within the past 3 months, experienced suicidal tendencies within the past 3 months, had an unstable or serious physical illness, pregnant or lactating women, and had a history or current thyroid disease or diabetes mellitus were excluded. In addition, BD patients who received treatment for thyroid disease or diabetes mellitus were also excluded since these therapies may affect glucose metabolism and the function of the HPA/HPT axis.

In this study, we also enrolled 119 age- and sex-matched healthy people as controls through an open form. All healthy controls were evaluated to rule out any current or past serious physical illnesses or mental disorders according to the International Classification of Diseases-10 (ICD-10).

### 2.3 Treatment

All enrolled patients received at least one mood stabilizer (including lithium or anti-epileptic, including lamotrigine, valproate, carbamazepine, and oxazepine) or one second-generation antipsychotic (olanzapine, quetiapine, aripiprazole, risperidone, or clozapine) with a stabilizing effect on mood. Some patients received other medications, such as antidepressants (including escitalopram, fluoxetine, paroxetine, sertraline, venlafaxine, fluvoxamine, citalopram hydrobromide, or duloxetine) and benzodiazepines (including clonazepam, lorazepam, alprazolam, oxazepam, or diazepam). The treatments provided are described in more detail in Appendix 2.

### 2.4 Evaluation indices

The general sociodemographic and clinical characteristics were collected through a questionnaire designed by the investigator. The Montgomery and Asberg Depression Rating Scale (MADRS) (26) and the Young Mania Rating Scale (YMRS) (27) were used to measure the BD clinical symptoms. The percentage change in the symptom score from baseline was calculated to assess the treatment response. The treatment was considered to be ineffective if the patients had less than a 25% reduction in symptoms at week 12 after treatment. Patients who had between 25% and 50% reduction in symptoms were deemed to be in remission, while those with a reduction in symptoms of 50% or more were deemed to be in significant remission (28). We defined an adverse course as three or more episodes of depressive or manic BD episodes before enrollment in our study, and two or fewer episodes were defined as non-adverse.

Adverse events were recorded at each follow-up and coded using the Treatment Emergent Symptom Scale (TESS) (29).

All blood samples were collected at 08:00 AM after an overnight fast, and the hormone levels were measured at certified laboratories located within the enrolled hospital.

The HPA axis hormones (adrenocorticotropic hormone (ACTH) and cortisol (CORT)) and HPT axis hormones (thyroid stimulating hormone (TSH), triiodothyronine (T3), thyroxine (T4), free triiodothyronine (fT3), and free thyroxine (fT4)) were measured at baseline and 12 weeks of post-treatment. The TSH, T3, fT3, T4, fT4, and CORT were determined by electrochemiluminescence (30), and ACTH was determined by radioimmunoassay (31). The normal ranges of the aforementioned neuroendocrine hormones were defined according to the West China Hospital of Sichuan University protocol as follows: TSH: 0.27–4.2 mu/L, fT3: 3.60–7.50 pmol/L, TT3: 1.3–3.1 nmol/L, fT4: 12–22 pmol/L, TT4: 62–164 nmol/L, ACTH: 5–78 ng/L, and CORT: 133–537 nmol/L. The biological data obtained from all study sites were converted to a unified unit and included in the analysis.

Glucose metabolism was assessed by the oral glucose tolerance test (OGTT), and the glycosylated hemoglobin was also measured. Insulin resistance was assessed by measuring the fasting blood glucose (FBG) and fasting serum insulin (FINS). The homeostasis model equation of insulin resistance (HOMA-IR), HOMA-IR = FBG (mmol/L)*FINS (mU/mL)/22.5, was used to calculate insulin resistance. Since some second-generation antipsychotics could impact glucose metabolism, we assessed the efficacy of these drugs by comparing changes in glucose metabolism between baseline and endpoint for the different treatments. The glucose metabolism and neuroendocrine abnormalities were classified as follows:

1. Increased thyroid hormone secretion (ISTH) defined as low TSH and/or high T3, fT3, T4, and/or fT4.
2. Decreased thyroid hormone secretion (DSTH) defined as high TSH and/or low T3, fT3, T4, and/or fT4
3. Increased secretion of ACTH and/or CORT (ISAC)
4. Decreased secretion of ACTH and/or CORT (DSAC)
5. Increased glucose metabolism (AGM-I) defined as the presence of impaired fasting glucose, abnormal glucose regulation, and/or diabetes.
6. Decreased glucose metabolism (AGM-D) defined as low FBG or 2-h postprandial blood glucose (2hPBG)
7. Insulin resistance (IR), which was defined as HOMA-IR greater than 2.0 (32).

The frequency of patients with specific neuroendocrine and glucose metabolism abnormalities was calculated for each BD group.

### 2.5 Data collection process

The investigators such as graduate students in psychiatry were trained to complete the symptom assessments. None of the investigators was involved in treatment delivery.

**Figure 1:**
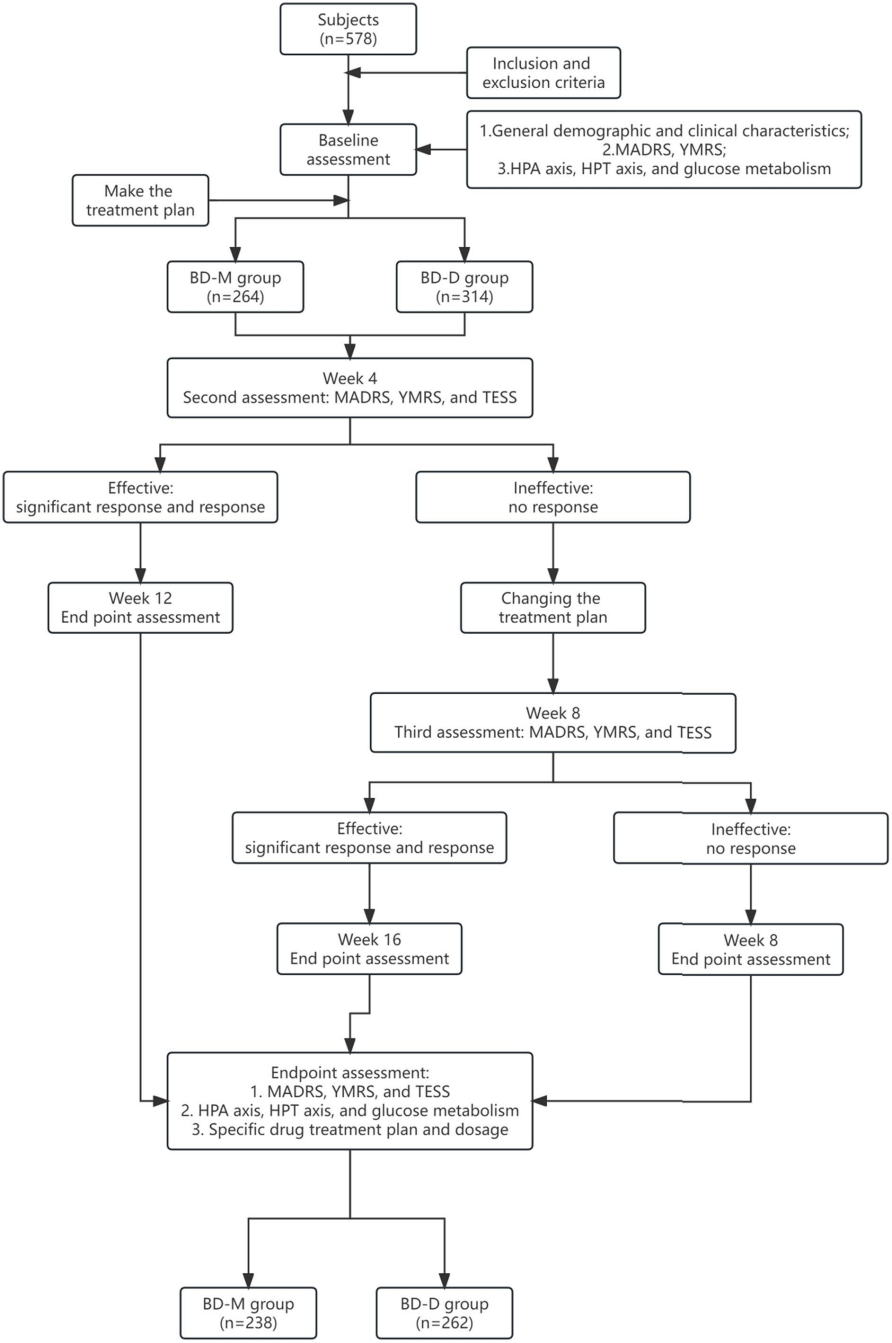
Flowchart illustrating the patient enrollment process.

The study process is described in more detail in Figure 1. Before treatment, the investigators assessed the clinical symptoms using the MADRS and YMRS tests. After 4 weeks of treatment, the investigators assessed any changes in the clinical symptoms using the MADRS and YMRS scores. In addition, the Clinical Global Impression (CGI) score was used to assess treatment efficacy and the TESS was used to assess the adverse reactions. If after 4 weeks the patient achieved a response to treatment, the same treatment was continued for an additional 8 weeks and the patient was reassessed. However, if the patients failed to respond to treatment, a new treatment regime was used. If the new treatment resulted in an effective response, the treatment was continued for another 8 weeks, and the endpoint was reached at week 16. If the patient still did not respond to treatment, the endpoint was reached at week 8.

### 2.6 Ethical considerations

All procedures performed in this study involving human participants were conducted in accordance with the Declaration of Helsinki (as revised in 2013). The study was approved by the Institutional Ethics Committee of the West China Hospital, Sichuan University [No. ChiECRCT-20180187], and registered on the chictr.org.cn database (date of first registration: October 24, 2018; identifier: ChiCTR1800019064). Written informed consent to participate in this study was obtained directly from all patients enrolled in the study. If the patient was not able to provide consent for this study, written informed consent was obtained from the legal guardian such as parents. More details about the consent procedure are provided in Figure 1.

### 2.7 Statistical analysis

All analyses were performed using the Statistical Package for Social Sciences (SPSS) version 26.0. The continuous data were summarized as mean ± standard deviation, while the categorical variables were expressed as percentages. A 2-sided Chi-square test was used to compare the categorical variables. The t-test or its non-parametric alternative was used to compare the hormone levels between the BD patients and the healthy individuals. The one-way analysis of variance (ANOVA) with Bonferroni correction (*p*_b_ = 0.05/number of tests) was used to analyze the difference between the healthy control, BD-D, and BD-M groups. The Pearson correlation analysis was used to determine the correlation between neuroendocrine levels, glucose metabolism, and the total MADRS and YMRS scores. The 95% confidence interval (95% CI) for each score was calculated using bootstrapping based on age stratification.

Binary logistic regression analysis adjusted for age and sex (forwards: LR) was used to calculate the association between adverse course (non-adverse = 1, adverse = 0) as the dependent variable and baseline neuroendocrine hormones and glucose metabolism levels as the independent variables. Similarly, the binary logistic regression analysis (forwards: LR) was used to assess the association between treatment effectiveness (effective = 1, ineffective = 0) as the dependent variable and the baseline clinical features reflecting disease severity, adverse course, neuroendocrine hormone levels, and glucose metabolism indicators as the independent variables.

For all statistical tests, a *p*-value below 0.05 was considered statistically significant. Patients with missing values were not included in the analyses.

## 3 Results

### 3.1 Demographics and clinical characteristics

A total of 578 patients were enrolled in the study, of whom 500 completed all of the above assessments and follow-ups. The drop-out rate was 13.49%. Out of the 500 enrolled patients, 238 were diagnosed with BD-M (mean age 36.70 years ± 12.78) and 262 patients were diagnosed with BD-D (mean age 37.18 years ± 14.04). In addition, 119 subjects were enrolled in the control group (mean age 38.48 years ± 9.37). The demographic and clinical characteristics of the participants are summarized in Table 1.

**Table 1:** The baseline demographic and clinical characteristics.

Characteristics		Mean ± standard deviation/patients, no. (%)				
		Total (*N* = 500)	BD-M group (*n* = 238)	BD-D group (*n* = 262)	Control group (*n* = 119)	*F*,*t*,*χ*^2^/*p*
Age (Y)		36.95 ± 13.43	36.70 ± 12.78	37.18 ± 14.04	38.48 ± 9.37	*0.671/0.512*
Sex	Male	246 (49.2%)	120 (50.4%)	126 (48.1%)	61(50.8%)	*0.300/0.861*
	Female	254 (50.8%)	118 (49.6%)	136 (51.9%)	58(48.3%)	
Nationality	Han	462 (92.4%)	232 (97.5%)	230 (87.7%)	*115(96.6%)*	*12.660/0.002*
	Ethnic minority	38 (7.6%)	6 (2.5%)	32 (12.3%)	*4(3.4%)*	
Address	City	292 (58.4%)	120 (50.4%)	172 (65.6%)	–	*5.952/0.015*
	Countryside	208 (41.6%)	118 (49.6%)	90 (34.4%)	–	
Educational level	Illiteracy	22 (4.4%)	12 (5.0%)	10 (3.8%)	–	*13.426/0.037*
	Primary school	54 (10.8%)	38 (16.0%)	16 (6.1%)	–	
	Junior high school	130 (26.0%)	72 (30.2%)	58 (22.2%)	–	
	Senior high school	102 (20.4%)	38 (16.0%)	64 (24.4%)	–	
	Junior college	88 (17.6%)	32 (13.4%)	56 (21.4%)	–	
	Bachelor	92 (18.4%)	38 (16.0%)	54 (20.6%)	–	
	Postgraduate or above	12 (2.4%)	8 (3.4%)	4 (1.5%)	–	
Marriage	Unmarried	174 (34.8%)	96 (40.3%)	78 (29.8%)	–	*4.620/0.329*
	Married	272 (54.4%)	120 (50.4%)	152 (58.0%)	–	
	Divorced	54 (10.8%)	22 (9.3%)	32 (12.2%)	–	
Occupation	Unemployed (including retirement)	140 (28.0%)	70 (29.4%)	70 (26.7%)	–	*16.443/0.021*
	Student or employed	360 (92.0%)	168 (70.6%)	192 (73.3%)	–	
Age at first onset, years		29.31.91 ± 11.79	27.58 ± 10.30	30.91 ± 12.84	–	*−2.238/0.025*
No. of episodes		3.56 ± 3.579	4.25 ± 4.21	2.78 ± 3.09	–	*2.672/0.008*
Time required for diagnosis (month)		30.37 ± 50.96	34.14 ± 49.36	26.57 ± 52.46	–	*1.140/0.256*
Family history	No	203 (81.2%)	99 (83.2%)	104 (79.4%)	–	*0.591/0.442*
	Yes	47 (18.8%)	20 (16.8%)	27 (20.6%)	–	
Total scores of MADRS		20.87 ± 13.17	13.15 ± 9.08	28.90 ± 11.92	–	*−10.593/0.000*
Total scores of YMRS		17.31 ± 16.91	29.15 ± 14.02	4.00 ± 7.16	–	*15.907/0.000*
Using lithium	No	436(87.2%)	202(84.9%)	234(89.3%)	–	*2.202/0.138*
	Yes	64(12.8%)	36(15.1%)	28(10.7%)	–	

MADRS, The Montgomery and Asberg Depression Rating Scale; YMRS, Young Mania Rating Scale.

### 3.2 Differences in neuroendocrine hormone secretion and glucose metabolism

The differences in the neuroendocrine hormone secretion and glucose metabolism between the BD patients and healthy individuals and the BD-M, BD-D, and healthy patients are summarized in Table 2.

**Table 2:** The baseline neuroendocrine/glucose metabolism characteristics.

Characteristics		Mean ± standard deviation/patients, no. (%)						
		Control group (*n* = 119)	Total (*n* = 500)	*t*/*p*^1^	BD-M group (*n* = 238)	BD-D group (*n* = 262)	*F*	*p* ^2^
HPA axis	CORT (nmol/L)	*385.98* ± 107.47	299.85 ± 287.49	3.099/0.002	294.22 ± 269.99	302.66 ± 297.66	*8.257*	*<0.001*
	ACTH (ng/L)	*24.48* ± 11.18	32.59 ± 25.48	−2.933/0.004	25.76 ± 14.44	35.88 ± 28.85	*4.629*	*0.011*
HPT axis	TSH (mU/L)	*2.30* ± 1.41	3.69 ± 6.47	3.073/0.002	4.29 ± 7.72	2.85 ± 2.44	*3.779*	*0.024*
	T3 (nmol/L)	*1.65* ± 0.25	1.68 ± 0.56	0.636/0.525	1.88 ± 0.61	1.54 ± 0.47^a^	*15.269*	*<0.001*
	fT3 (pmol/L)	*5.00* ± 0.73	4.76 ± 1.37	−2.153/0.032	5.09 ± 1.44	4.50 ± 1.25^a^	*8.684*	*<0.001*
	T4 (nmol/L)	*95.69* ± 15.52	83.87 ± 36.46	−4.147/0.000	95.15 ± 37.09	75.64 ± 33.83^a^	*17.447*	*<0.001*
	fT4 (pmol/L)	*17.02* ± 3.14	15.01 ± 6.99	−3.644/0.000	16.13 ± 5.77	14.15 ± 7.72	*7.569*	*0.001*
	fT3/fT4 ratio	*0.30* ± 0.05	0.57 ± 0.89	−23.291/0.000	0.48 ± 0.80	0.63 ± 0.98	*6.351*	*0.002*
Glucose metabolic states	FBG (nmol/L)	*4.73* ± 0.40	5.17 ± 1.34	4.799/0.000	5.35 ± 1.29	5.02 ± 1.37	*1.888*	*0.152*
	2hPBG (nmol/L)	6.46 ± 1.01	7.58 ± 2.88	5.485/0.000	7.88 ± 3.47	7.32 ± 2.18	*1.179*	*0.309*
	FINS (μU/mL)	6.63 ± 2.74	9.70 ± 6.33	5.491/0.000	9.16 ± 6.26	10.23 ± 6.38	*0.582*	*0.559*
	HOMA-IR	1.41 ± 0.66	2.20 ± 1.48	6.117/0.000	2.14 ± 1.51	2.29 ± 1.45	*0.223*	*0.800*
	HbA1c (%)	5.3 ± 0.35	5.22 ± 0.69	1.537/0.125	5.12 ± 0.82	5.30 ± 0.55	*1.924*	*0.147*

HPA, hypothalamic–pituitary–adrenal; HPT, hypothalamic–pituitary-thyroid; TSH, thyroid-stimulating hormone; T3, total triiodothyronine; fT3, free triiodothyronine; T4, total thyroxine; fT4, free thyroxine; CORT, cortical hormone; ACTH, adrenocorticotrophic hormone; FBG, fasting blood glucose; 2hPBG, 2-h postprandial blood glucose; FINS, fasting insulin; HbA1c, glycosylated hemoglobin; HOMA-IR, homeostasis model equation; *N*, number.

*p^1^ represents the differences between the control and the total group. p^2^ represents the differences between the control, BD-M, and BD-D groups*.

After Bonferroni correction, *p* = 0.05/6 was used for the HPA axis, *p* = 0.05/18 was used for the HPT axis, and *p* = 0.05/15 was used for the glucose metabolism states.

a Represents a significant difference compared to the BD-M group in pairwise comparison.

At baseline, the ACTH and CORT levels did not differ significantly between the BD-D and BD-M groups (*p*_ACTH_ = 0.011, *p*_CORT_ = 0.846). However, the BD-D group had significantly higher ACTH levels than the control group (*p* < 0.001) even after applying the Bonferroni correction (*p*_b_ = 0.008). Conversely, the BD-M group had significantly higher T3, fT3, and T4 (all *p* < 0.001) levels than the BD-D group. Similarly, the T3, T4, and fT4 levels in the BD-M group were significantly higher than those in the control group (*p* < 0.001). Compared to the BD-M, the fT3/T4 levels were significantly lower than those in the BD-D group, while the fT3/T4 levels in the control group were lower than those of all BD patients combined (*p* < 0.001).

There were no significant differences in glucose metabolism between the BD-D and BD-M groups.

### 3.3 Frequency of abnormal neuroendocrine hormone secretions and glucose metabolism

The frequency of abnormal neuroendocrine hormone secretions and glucose metabolism in patients with BD-M and BD-D is summarized in Table 3. Since studies have found an association between lithium and hypothyroidism (33), we also measured the proportion of DSTH in patients who were not treated with lithium. The proportions of DSTH in the BD-M and BD-D groups were 32.7% (66/202) and 41.9% (98/234), respectively. No significant differences in the incidence of abnormal neuroendocrine hormone secretion and glucose metabolism were noted between the two groups, irrespective of the treatment provided.

**Table 3:** The incidence of abnormal hormone secretion and glycometabolism between the BD-M and BD-D groups.

	ISTH	DSTH	ISAC	DSAC	AGM-I	AGM-D	IR
BD-M	6.7% (16/238)	38.7% (92/238)	10.5% (25/238)	26.1% (61/238)	45.4% (108/238)	5.9% (14/238)	45.8% (109/238)
BD-D	4.6% (12/262)	45% (118/262)	23.6% (62/262)	28.2% (74/262)	37.4% (98/262)	11.5% (30/262)	49.2% (129/262)
*χ*^2^/*p*	*0.541/0.585*	*1.043/0.369*	*2.774/0.135*	*0.152/0.776*	*1.636/0.247*	*2.409/0.179*	*0.233/0.629*

ISTH, increased thyroid hormone secretion; DSTH, decreased thyroid hormone secretion; ISAC, increased secretion of ACTH and CORT; DSAC, decreased secretion of ACTH and CORT; AGM-I, abnormal glucose metabolism-increased; AGM-D, abnormal glucose metabolism-decreased; IR, insulin resistance.

### 3.4 Correlation analysis

In the BD-M group, the baseline YMRS scores were positively correlated with baseline T4 (*r* = 0.349, *p* = 0.010) and fT4 (*r* = 0.335, *p* = 0.013) and negatively correlated with the FINS (*r* = −0.289, *p* = 0.013). In the BD-D group, the baseline total MADRS scores were significantly positively correlated with the baseline fT3 (*r* = 0.223, *p* = 0.032) and fT4 (*r* = 0.315, *p* = 0.002).

### 3.5 Regression analysis

#### 3.5.1 Relationship between the HPT and HPA axes functions, glucose metabolism levels, and treatment outcomes

The mean reduction rate in the MADRS in the BD-D group was 51.70% ± 22.41% after 4 weeks and 75.66% ± 18.28% after 12 weeks. The mean reduction rate in the YMRS in the BD-M group was 52.63 ± 50.82 and 87.51% ± 17.41% during the same time course.

**Figure 2:**
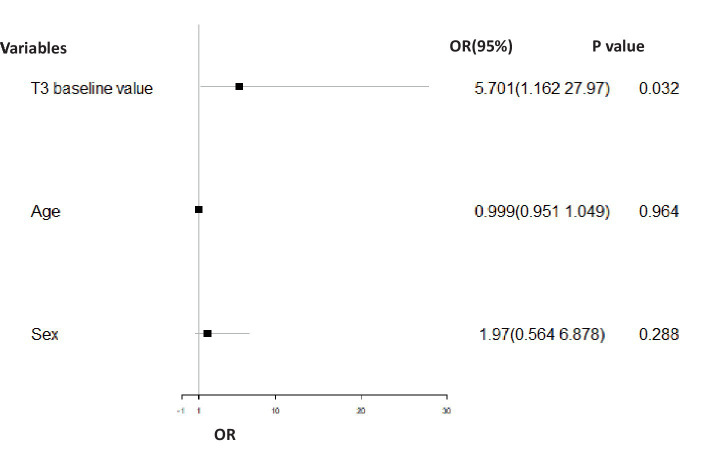
Binary logistic regression analysis comparing the odd ratio for effective treatment in the BD-D group adjusted for gender and age. In the BD-D group, the T3 baseline value (*p* = 0.032, OR = 5.071, 95% CI = 1.162–27.970) had a significant positive association with treatment effect (effective = 1, ineffective = 0) at 12 weeks of post-treatment.

For the BD-D group, the T3 baseline value (*p* = 0.032, OR = 5.071, 95% CI = 1.162–27.970) was significantly positively related to the treatment effect at 12 weeks after controlling for age and sex (Figure 2). For the BD-M group, important baseline clinical characteristics, neuroendocrine hormone, and glucose metabolism indices had no significant effect on the treatment effect at 12 weeks.

### 3.6 Relationship between the HPT and HPA axes functions and glucose metabolism levels and adverse disease course

**Figure 3:**
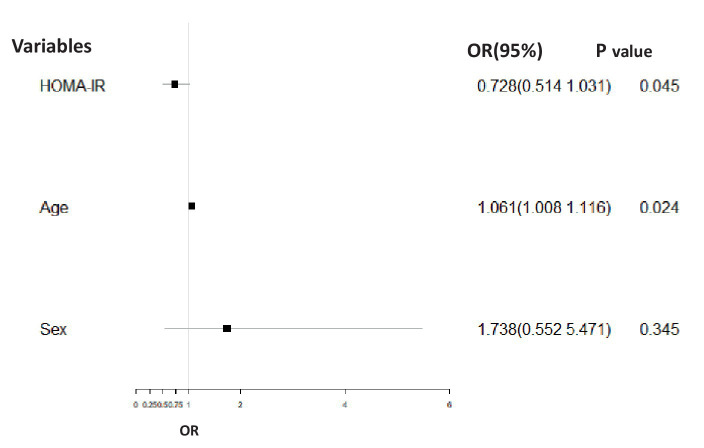
Binary logistic regression analysis corrected for gender and age comparing the association between the adverse course (non-chronic = 1, chronic = 0) and HOMA-IR in the BD-M group. The HOMA-IR baseline value (*p* = 0.045, OR = 0.728, 95% CI = 0.514–1.031) was significantly related to adverse disease course.

For the BD-M group, the HOMA-IR baseline value (*p* = 0.045, OR = 0.728, 95% CI = 0.514–1.031) was significantly related to the adverse course after controlling for age and sex (Figure 3).

### 3.7 Hormone secretion and glycometabolism differences between baseline and endpoint after psychotropic drug medication

A total of 26 subjects failed to complete the HPA axis detection (14 in the BD-D group and 12 in the BD-M group) and 32 subjects failed to complete the OGTT detection (12 in the BD-D group and 20 in the BD-M group) at the endpoint.

The results showed that the fT3/fT4 ratio (t = 2.966, *p* = 0.003) significantly decreased at the endpoint in the BD group. After 12 weeks of treatment, the T4 (t = 6.191, *p* = 0.014), T3 (t = 9.502, *p* = 0.002), and HbA1c (t = 4.062, *p* = 0.045) levels increased significantly, while the fT3/fT4 ratio (t = 2.834, *p* = 0.005) decreased significantly in the BD-D group. No significant difference was noted between the baseline and 12-week post-treatment HPT axis, HPA axis, and glucose metabolism indices in the BD group, the BD-D group, and the BD-M group (Table 4).

**Table 4:** Hormones secretion and glycometabolism differences between baseline and after 12 weeks of treatment.

Indicator		Total (*N* = 500)		BD-M (*n* = 238)		BD-D (*n* = 262)	
		Baseline	Endpoint	Baseline	Endpoint	Baseline	Endpoint
HPT axis	TSH (mU/L)	3.69 ± 6.47	3.68 ± 4.66	4.29 ± 7.72	4.78 ± 6.33	2.85 ± 2.44	2.71 ± 1.91
	T3 (nmol/L)	1.68 ± 0.56	1.79 ± 0.63	1.88 ± 0.61	1.88 ± 0.66	1.54 ± 0.47	1.72 ± 0.59*
	fT3 (pmol/L)	4.76 ± 1.37	4.94 ± 5.10	5.09 ± 1.44	5.33 ± 7.40	4.50 ± 1.25	4.59 ± 0.80
	T4 (nmol/L)	83.87 ± 36.46	89.61 ± 24.62	95.15 ± 37.09	90.94 ± 27.89	75.64 ± 33.83	88.25 ± 21.24**
	fT4 (pmol/L)	15.01 ± 6.99	15.32 ± 4.34	16.13 ± 5.77	15.60 ± 5.03	14.15 ± 7.72	15.08 ± 3.64
	fT3/fT4 ratio	0.57 ± 0.89	0.36 ± 0.40**	0.48 ± 0.80	0.37 ± 0.41	0.63 ± 0.98	0.36 ± 0.40**
HbA1c (%)		5.22 ± 0.69	5.34 ± 0.77	5.12 ± 0.82	5.23 ± 0.83	5.30 ± 0.55	5.49 ± 0.72*

		Total (*N* = 474)		BD-M (*n* = 226)		BD-D (*n* = 248)	
		Baseline	Endpoint	Baseline	Endpoint	Baseline	Endpoint
HPA axis^a^	CORT (nmol/L)	299.85 ± 287.49	275.96 ± 281.28	294.22 ± 269.99	199.07 ± 214.64	302.66 ± 297.66	216.79 ± 240.61
	ACTH (ng/L)	32.59 ± 25.48	30.55 ± 16.27	25.76 ± 14.44	28.89 ± 16.19	35.88 ± 28.85	31.48 ± 16.42

		Total (*N* = 468)		BD-M (*n* = 218)		BD-D (*n* = 250)	
		Baseline	Endpoint	Baseline	Endpoint	Baseline	Endpoint
OGTT^b^	FBG (nmol/L)	5.17 ± 1.34	5.18 ± 1.33	5.35 ± 1.29	5.33 ± 1.65	5.02 ± 1.37	4.99 ± 0.84
	2hPBG (nmol/L)	7.58 ± 2.88	7.33 ± 2.32	7.88 ± 3.47	7.66 ± 2.51	7.32 ± 2.18	6.99 ± 2.05
	FINS (μU/mL)	9.70 ± 6.33	9.08 ± 8.55	9.16 ± 6.26	7.46 ± 3.93	10.23 ± 6.38	11.22 ± 11.98
	HOMA-IR	2.20 ± 1.48	2.15 ± 2.04	2.14 ± 1.51	1.81 ± 1.18	2.29 ± 1.45	2.60 ± 2.75

a The data were missing for 26 cases, including 14 in the BD-D group and 12 in the BD-M group.

b The data were missing in 32 cases including 12 in the BD-D group and 20 in the BD-M group.

**p < 0.01; *p < 0.05.

HPA, hypothalamic–pituitary–adrenal; HPT, hypothalamic–pituitary-thyroid; OGTT, oral glucose tolerance test; TSH, thyroid-stimulating hormone; T3, total triiodothyronine; fT3, free triiodothyronine; T4, total thyroxine; fT4, free thyroxine; CORT, cortical hormone; ACTH, adrenocorticotrophic hormone; FPG, fasting plasma glucose; 2hPBG, 2-h postprandial blood glucose; FINS, fasting insulin; HbA1c, glycosylated hemoglobin; HOMA-IR, homeostasis model equation; *N*, number.

## 4 Discussion

Optimized drug management plays a crucial role in stabilizing mood, preventing relapses, and improving the overall quality of life for individuals with bipolar disorder. As a result, there is a need to identify optimal biomarkers to assess treatment response and, hence, guide treatment interventions. Although studies found an association between abnormalities in the HPA, HPT, and glucose metabolism indices and the onset of depressive disorders, the exact association between these indices and treatment response in BD-D and BD-M patients remains unclear. In this study, we examined the characteristics of the HPA and HPT axis functions and glucose metabolism in BD patients in relation to healthy controls. In addition, we also evaluated the association between the HPA, HPT, and glucose metabolism indices on treatment outcomes in patients with BD-M and BD-D.

In our study, irrespective of the BD-D and BD-M severity, no abnormalities were detected in the secretion of ACTH and CORT in the BD-D and BD-M groups. These findings are consistent with the results of a previous Chinese study (34). However, other studies found an association between HPA axis dysfunction and the onset of depression, especially in patients with major depressive disorders (35, 36). The HPA axis is an important mediator of the stress response and may, therefore, have an important role in the onset of depressive disorders (37). In addition, we also found no significant differences in the ACTH and cortisol levels between the BD-D and BD-M groups. These findings indicate that although HPA axis dysfunction might lead to the development of BD, which does not affect the disease state. Previous studies have shown that dysregulation of ACTH and cortisol occurs in response to CRH stimulation in BD patients, especially in patients with BD-D or cyclical BD (38, 39). However, for patients with BD-M, the changes in CRH secretion seem to appear before the onset of the hypomanic symptoms and the patients exhibit normal cortisol suppression (37). Therefore, the HPA axis indices could potentially be used as a marker of BD (37).

Accumulating evidence suggests that HPT axis dysfunction may contribute to the pathophysiology and clinical course of BD (12). HPA dysfunction could occur through negative regulation of TSH secretion mediated by ACTH signaling (8). In this study, a high proportion of patients in both BD-D and BD-M groups suffered from thyroid dysfunction, especially hypothyroidism. These findings remained consistent even after removing patients treated with lithium, which is a drug known for triggering hypothyroidism. Consistent with previous studies (9, 40), patients in the BD-D group had significantly lower T3, fT3, and T4 levels when compared to patients in the BD-M group. However, no difference was found in the TSH levels between two groups. These results indicate that the HPT axis dysfunction tends to differ in patients with BD-D and BD-M. BD patients with subclinical hypothyroidism also tend to have deficits in verbal memory, attention, language, and executive function (41). Therefore, thyroid function tests particularly the monitoring of T3 and T4 levels could be used to exclude thyroid dysfunction in BD patients and to monitor treatment response.

However, there was no significant correlation between TSH and the severity of bipolar depression and bipolar mania, which is consistent with previous studies (42). Consistent with previous works, we also found a significant positive correlation between baseline T4 and fT4 and the severity of BD-M (43–46). Elevated T4 or fT4 has also been reported during the acute phase of psychiatric disorders (47), as well as during manic BD episodes. This increased T4 activity may reflect a protective compensatory homeostatic mechanism to maintain a normal brain function during the acute phase (47). Elevated T4 levels may also occur in response to stress induced by hospitalization or changes in the physiological state. Previous studies have found that T4 or fT4 elevation is transient and gradually returns to normal after a few weeks of treatment (48). Indeed, the rate of T4 decline is closely associated with good prognosis (49), and T4 supplementation (50) is often used to treat BD. Therefore, T4 or fT4 could be used as potential biomarkers to assess the disease severity and treatment response in BD patients during the acute manic state. However, further studies are required to identify the mechanisms that lead to an increase in the T4 or fT4 levels at the beginning of treatment and their rapid decline after treatment.

Studies evaluating the relationship between TSH, T3, and T4 dysregulation and the development of major depressive disorders and bipolar depression revealed conflicting results (42). These differences could be related to the fact that in unipolar depression, hypothyroidism occurs due to dysfunction in the HTA axis (51), whereas BD-D dysregulation of the HTA axis may occur as a result of a lack of response to thyroid hormones in peripheral tissues (50). The fT3/fT4 ratio can better reflect changes in the peripheral activity of thyroid hormones. In our study, we noted a significant decrease in the fT3/fT4 ratio in the BD-D group at the end of the acute phase. Although some studies have reported that higher thyroid hormones were associated with mania, some studies reported an association between hyperthyroidism and depression (52). Overall, these findings indicate a complex relationship between thyroid dysfunction and psychiatric disorders.

Similar to previously published studies, we also noted that the baseline total MADRS scores of the BD-D group were significantly positively correlated with fT3 and fT4 levels, which is consistent with prior studies (34, 53). Recent studies have shown that reduced TSH levels were correlated with disrupted intrinsic activity of the precuneus (PCu)/posterior cingulate cortex (PCC) in BD patients compared to healthy controls (54), and the HPT axis dysfunction might be implicated in cognitive impairment in the early stages of BD (55). However, other studies suggest that patients with a lower average pretreatment-free thyroxine index have more serious depressive symptoms (56). The difference in these studies may be due to the proportion of subjects treated with lithium. The T3 baseline value was also significantly positively related to the treatment effect at 12 weeks in the BD-D group after adjusting for age and sex. In the acute bipolar depressive state, elevated fT3 and fT4 levels were associated with more rapid treatment response, shorter hospital stay, lower rate of repeated BD episodes, and improved glucose metabolism (57, 58). These findings could be attributed to better regulation of the anterior limbic neural network, especially the thalamus, striatum, and subgenual cortex in patients with elevated fT3 and fT4 (50). Excessive activation of the anterior limbic neural network in BD patients may alter the positive and negative feedback loops responsible for the regulation of emotions (59). Conversely, a decline in the fT3 and fT4 in the acute bipolar depressive state may indicate an early decline in the thyroid function, leading to poor treatment response (58, 60). Therefore, T3, fT3, and fT4 levels could potentially be used as biomarkers for disease severity and treatment response in the acute BD-D state. Therefore, these indicators may help physicians to determine the thyroid function, especially in patients with poor response to lithium therapy.

Consistent with previous studies (61), we also found a high incidence of AGM-I and insulin resistance in both the BD-M and BD-D groups. The HOMA-IR baseline value was significantly related to adverse disease course after adjusting for age and sex in the BD-M group, but not in the BD-D group. Previous studies also found that insulin resistance may also play an important role in the development of treatment resistance in BD patients (56), after controlling for medication and body mass index. The hyperactivation of the HPA axis is known to elevate oxidative stress levels and disrupt mitochondrial energy metabolism, particularly in the context of insulin dysmetabolism. This disturbance can contribute to an augmented allostatic load in individuals with BD and may potentially contribute to disease progression and a more pronounced neurological deficit (62, 63) due to alterations in the brain energy metabolism and structure (20, 21). A systematic literature review (64) showed that comorbid insulin resistance might also contribute to the development of early cognitive impairment in BD patients. This cognitive impairment often presents as a gradual decline in verbal communication, numerical reasoning ability, and mental processing speed (62). Lithium administration may have some positive effects on the treatment outcomes of BD patients with comorbid insulin resistance. Lithium has an important role in the regulation of the GSK3β/AKT pathway, which is known to be a key player in the regulation of insulin signaling pathways. As a result, the regular monitoring of glucose metabolism in BD patients could facilitate the early detection of patients with insulin-resistant BD, which leads to poor glucose metabolism and brain structural damage, thus allowing the therapist to optimize treatment for this category of patients (20, 21, 65–66). However, further longitudinal studies are required to identify the feasibility of using glucose metabolism as a biomarker for insulin resistance following lithium therapy.

Another interesting finding was that the patients in the BD-M group were more likely to have the first onset of the disease at younger age, had a high frequency of BD episodes, were less educated, and were more likely to be unemployed and live in rural residents than patients in the BD-D group. This finding suggests that patients with BD-M are more likely to have a worse disease burden than those with BD-D as the manic episodes tend to cause more disruption to patient’s life (67).

This study has some limitations that have to be acknowledged. The short 12-week follow-up period only allowed us to observe the treatment effect during the acute phase of the disease. In addition, the hormone indicators were collected at a single time point throughout the day and, therefore, these measurements did not reflect the hormonal changes that occur as a result of the circadian rhythm. Moreover, due to the limited number of patients with cyclical BD and in the remission state, we could not include them in the study. Some factors that might interfere with hormone changes or glucose metabolism status, such as smoking, body mass index, menopausal status, lifestyle, treatment modalities, and recruitment sites, were not taken into account in our study. We also excluded patients with recent substance dependence and suicidal tendencies to reduce the risk of introducing a selection bias. Finally, since this study was conducted in Western China, the generalizability of these findings might be limited.

## 5 Conclusion

Although the HPT axis dysfunction varied between patients with BD-M and BD-D, no significant differences in the HPA axis dysfunction and glucose metabolism were noted between two groups. The HPT axis function and glucose metabolism were closely associated with clinical outcomes and disease course during the acute BD phase. Continuous monitoring of HPA, HPT, and glucose metabolism may provide important biological information on the severity of the disease and treatment outcomes. More specifically, the monitoring of T3 in BD-D patients and HOMA-IR for BD-M patients could provide the most valuable insight into treatment response. However, further longitudinal studies with cognitive function tests and functional brain magnetic resonance imaging are required to confirm the impact of these hormones on disease progression.

## Data availability statement

The original contributions presented in the study are included in the article/Supplementary material, further inquiries can be directed to the corresponding authors.

## Ethics statement

The studies involving humans were approved by Institutional Ethics Committee of the West China Hospital, Sichuan University. The studies were conducted in accordance with the local legislation and institutional requirements. The participants provided their written informed consent to participate in this study. Written informed consent was obtained from the individual(s) for the publication of any potentially identifiable images or data included in this article.

## Author contributions

XZ: Formal analysis, Methodology, Writing – original draft, Writing – review & editing. YZ: Formal analysis, Investigation, Writing – original draft. YC: Formal analysis, Investigation, Writing – original draft. SZ: Data curation, Formal analysis, Investigation, Writing – original draft. BZ: Conceptualization, Project administration, Supervision, Writing – original draft. XS: Conceptualization, Funding acquisition, Methodology, Project administration, Supervision, Writing – review & editing.
