# Supplementary material for: The association between neuroendocrine/glucose metabolism and clinical outcomes and disease course in different clinical states of bipolar disorders
Source: Front Psychiatry. 2024 Jan 24;15:1275177. doi: 10.3389/fpsyt.2024.1275177 (PMC10847283; doi:10.3389/fpsyt.2024.1275177)
Supplement: Supplementary file 1 [file Data_Sheet_1.PDF]

Appendix 1: study sites in the real-world study from December,31,2018 to December,31,2019.

Name of The Sites
West China Hospital of Sichuan University
The First Affiliated Hospital of Chongqing Medical University
Zhujiang Hospital Affiliated to Southern Medical University
Affiliated Hospital of Southwest Medical University
Affiliated Hospital of North Sichuan Medical College
Sichuan Provincial People's Hospital
Mianyang Third People's Hospital
Deyang people's Hospital
Guangyuan mental health center
Zigong mental health center
Panzhihua Third People's Hospital
Guangxi Zhuang Autonomous Region Brain Hospital
Tianshui Third People's Hospital
Yunnan mental health center
Urumqi Fourth People's Hospital

Appendix 2:The list of medications used in each group

BPD-D group(n=262)		BPD-M group(n=238)	
Treatment models (Patients, No.)	Patients, No. (%)	Treatment models (Patients, No.)	Patients, No. (%)
Triple-therapy(n=142)		Triple-therapy(n=94)	
MS		MS	
Valproate	74(52.1%)	Valproate	46(48.9%)
Lamotrigine	34(23.9%)	Lithium	22(23.4%)
Lithium	21(14.8%)	Carbamazepine	9(9.6%)
Oxazepine	7(4.9%)	Lamotrigine	9(9.6%)
Carbamazepine	6(4.2%)	Oxazepine	8(8.5%)
SGA		SGA	
Olanzapine	53(37.3%)	Olanzapine	51(54.3%)
Quetiapine	38(26.8%)	Quetiapine	21(22.3%)
Risperidone	29(20.4%)	Risperidone	12(12.8%)
Aripiprazole	22(15.5%)	Aripiprazole	10(10.6%)
AD		AD	
Paroxetine	43(30.3%)	Fluoxetine	33(35.1%)
Escitalopram	31(21.8%)	Venlafaxine	19(20.2%)
Fluoxetine	28(19.7%)	Paroxetine	11(11.7%)
Sertraline	17(12.0%)	Sertraline	11(11.7%)
Venlafaxine	8(5.6%)	Fluvoxamine	10(10.6%)
Fluvoxamine	7(4.9%)	Escitalopram	10(10.6%)
Citalopram hydrobromide	4(2.8%)		
Duloxetine	4(2.8%)		
Double-therapy(n=99)		Double-therapy(n=133)	
MS(n=60)		MS(n=108)	
Valproate	43(71.7%)	Valproate	99(91.7%)
Lithium	11(18.3%)	Lamotrigine	5(4.6%)
Lamotrigine	6(10.0%)	Lithium	4(3.7%)
SGA(n=65)		SGA(n=133)	

Quetiapine	33(50.7%)	Olanzapine	54(40.6%)
Olanzapine	17(26.2%)	Quetiapine	35(26.3%)
Risperidone	8(12.3%)	Risperidone	26(19.6%)
Aripiprazole	5(7.7%)	Aripiprazole	14(10.5%)
Clozapine	2(3.1%)	Clozapine	4(3.0%)
AD(n=72)		AD(n=25)	
Sertraline	23(31.9%)	Venlafaxine	16(64.0%)
Paroxetine	15(20.8%)	Fluoxetine	6(24.0%)
Venlafaxine	11(15.3%)	Fluvoxamine	3(12.0%)
Fluoxetine	8(11.1%)		
Escitalopram	8(11.1%)		
Duloxetine	3(4.2%)		
Citalopram hydrobromide	3(4.2%)		
Fluvoxamine	1(1.4%)		
Monotherapy(n=21)		Monotherapy(n=11)	
MS(n=12)		MS	
Lithium	4(33.2%)	Valproate	6(54.5%)
Lamotrigine	2(16.7%)	Lithium	2(18.2%)
Valproate	2(16.7%)	Carbamazepine	1(9.1%)
Oxazepine	2(16.7%)	Lamotrigine	1(9.1%)
Carbamazepine	2(16.7%)	Oxazepine	1(9.1%)
SGA(n=9)			
Olanzapine	4(44.5%)		
Quetiapine	2(22.2%)		
Aripiprazole	1(11.1%)		
Risperidone	1(11.1%)		
Clozapine	1(11.1%)		

Abbreviations: MS, mood stabilizers; SGA, second generation antipsychotic; AD, antidepressants. All the kinds of drugs were used within the dosage range specified in the drug specification.
